# Colonization, penetration and transformation of manganese oxide nodules by *Aspergillus niger*

**DOI:** 10.1111/1462-2920.14591

**Published:** 2019-04-02

**Authors:** John Ferrier, Yuyi Yang, Laszlo Csetenyi, Geoffrey Michael Gadd

**Affiliations:** ^1^ Geomicrobiology Group School of Life Sciences, University of Dundee Dundee, DD1 5EH Scotland, UK; ^2^ Concrete Technology Group, Department of Civil Engineering University of Dundee Dundee, DD1 4HN Scotland, UK

## Abstract

In this study, the ability of the geoactive fungus *Aspergillus niger* to colonize and transform manganese nodules from the Clarion‐Clipperton Zone in both solid and liquid media was investigated. *Aspergillus* *niger* was able to colonize and penetrate manganese nodules embedded in solid medium and effect extensive transformation of the mineral in both fragmented and powder forms, precipitating manganese and calcium oxalates. Transformation of manganese nodule powder also occurred in a liquid medium in which *A. niger* was able to remove the fine particles from suspension which were accumulated within the central region of the resulting mycelial pellets and transformed into manganese oxalate dihydrate (lindbergite) and calcium oxalate dihydrate (weddellite). These findings contribute to an understanding of environmental processes involving insoluble manganese oxides, with practical relevance to chemoorganotrophic mineral bioprocessing applications, and, to the best of our knowledge, represent the first demonstration of fundamental direct and indirect interactions between geoactive fungi and manganese nodules.

## Introduction

Seafloor polymetallic manganese nodules have long been considered to be vital future resources of Ni, Mn and Cu and have received increased attention in recent years because of the significant concentrations of rare metals and rare earth elements (REEs) also hosted in these substrates (Hein *et al*., 2013). Nodules from the Clarion‐Clipperton Zone (CCZ) alone represent more Mn, Ni and Co than all terrestrial reserve bases and comprise Mn, 5992 million tonnes; Ni, 274 million tonnes and Co, 44 million tonnes (Hein *et al*., 2013; Hein, 2016).

Polymetallic manganese nodules are formed of concentric layers of both Mn‐oxides and Fe‐oxyhydroxides around a central nucleus. Minerals may precipitate from ions in the overlaying seawater through hydrogenetic growth processes, yielding oxides such as vernadite (δ‐MnO_2_). Additionally, nodule growth occurs through diagenetic processes, in which ions mobilized from sediment are supplied by pore waters, resulting in the precipitation of phyllomanganate (MnO_6_) minerals such as todorokite [(Na_0.2_Ca_0.05_K_0.02_)(Mn^4+^_4_, Mn^3+^_2_)O_12_∙3H_2_O] and birnessite [(Na_0.3_Ca_0.1_K_0.1_)(Mn^4+^ Mn^3+^)_2_O_4_·1.5H_2_O]. Vernadite and birnessite can exhibit high levels of isomorphic substitution of Mn^4+^ by alternative metal cations such as Ni^2+^ and Co^3+^ (Hein, 2016; Kuhn *et al*., 2017). Additionally, MnO_2_ surfaces have a high sorptive capacity for metal cations including Ni^2+^, Co^2+^ and Zn^2+^ (Miyata *et al.,* 2004; 2006). These processes combined with a high surface area and extremely slow growth rate (hydrogenetic growth can be as low as 1–5 mm per million years) contribute to the concentration over time of elements present as soluble cations in seawater (Hein, 2016; Kuhn *et al*., 2017).

Geoactive fungi such as *Aspergillus niger* occupy an important role in bioweathering processes and element cycling in the terrestrial environment (Fomina *et al.,* 2004; Adeyemi and Gadd, 2005). They are able to colonize mineral substrates and effect mineral transformations as a combined function of physical weathering from mechanical forces exerted by hyphae, and chemical weathering by, for example, the excretion of organic acids and other metabolites (Gadd, 2007; Rosling *et al*., 2009). This enables the fungi to etch, penetrate or deteriorate mineral substrates and enhances resulting bioweathering processes such as elemental cycling, mineral dissolution and mineral formation. The secretion of organic acids is of particular significance to biogeochemical processes due to their ability to leach metals from mineral substrates by proton‐mediated and ligand‐mediated dissolution, and also, in some cases, to precipitate them as insoluble biominerals, for example, oxalates (Fomina *et al*., 2005; Gadd *et al.,* 2014). The transformation of mineral substrates by organic acid producing fungi, including *A. niger,* is well established and a range of minerals have been investigated such as vanadinite, mimetite and gypsum (Gharieb *et al.,* 1998; Ceci *et al*. 2015a, b). Furthermore, the biotransformation of MnO_2_, Mn_2_O_3_, biogenic manganese oxides and birnessite by *A. niger* has also been demonstrated (Wei *et al.,* 2012). Manganese oxide minerals such as these are some of the strongest oxidants present in the environment. As such, they have a significant role in element cycling and redox reactions with a range of organic and inorganic substrates, in both terrestrial and aquatic settings. Furthermore, due to the high sorptive capacities of manganese oxide minerals, they are central to the distribution and availability of a wide range of elements (Miyata *et al.,* 2004; Tebo *et al.,* 2004; Miyata *et al.,* 2006; Learman *et al.,* 2011a, 2011b; Wei *et al.,* 2012).

While the biogeochemical significance of fungi is frequently perceived as being most evident in aerobic regions of the terrestrial environment such as aerobic soils and sediments, the plant root zone, and rock and mineral surfaces (Gadd, 2007), it is now known that fungi can be detected in anaerobic environments normally thought to be quite inhospitable for growth and activity (Orsi *et al*., 2013; Ivarsson *et al*., 2016a,b; Gadd 2017a,b; 2018). Such locations include the seafloor, deep sea sediments, igneous oceanic crust, hydrothermal vents and methane cold seeps (Reitner *et al*., 2006; Ivarsson, 2012; Bengtson *et al*., 2014; Ivarsson *et al*. 2016a,b). While the existence of anaerobic fungi, other than certain yeasts, has been known for a long time (Orpin, 1994; Gruninger *et al*., 2014; Ivarsson *et al*., 2016b), the occurrence of fungi in such habitats raises questions about their roles and interactions with other organisms and the substrate. While a symbiotic relationship with chemolithotrophic prokaryotes is important in the igneous oceanic crust, it seems that fungi are involved in a variety of mineral transformations including biomineralization and mineral dissolution (Ivarsson *et al*., 2016a,b). Biomineralized hyphae are frequently observed in deep sub‐surface locations (Reitner *et al*., 2006) and as well as penetration of carbonates and zeolites (Bengtson *et al*., 2014; Ivarsson *et al*., 2015). Fungi can also be associated with manganese oxides both in terms of formation through Mn(II) oxidation to Mn(IV) (Ivarsson *et al*., 2015) or properties of Mn(IV) dissolution (Wei *et al*., 2012).

The bioweathering capabilities of geoactive fungal species also suggest that they are suited to certain metal bioprocessing applications (Gadd, 2010). Several studies have examined the ability of various species to leach metals from a variety of substrates including low‐grade ores and residues (Mulligan and Galvez‐Cloutier, 2000; Mulligan *et al*., 2004; Biswas *et al*., 2013), fly ash (Wu and Ting, 2006; Xu and Ting, 2009; Xu et al., 2014) and electronic wastes (Jadhav *et al.,* 2016). Additionally, the capabilities of organic acids have also been studied for application in abiotic leaching processes (Strasser *et al*., 1994; Astuti *et al*., 2016).

The objective of this research was to investigate direct interactions between *A. niger* and Clarion‐Clipperton zone manganese nodules such as colonization, penetration and metal and mineral transformation. Although mineral bioprocessing has received increased attention in recent years, fundamental data do not exist regarding direct interactions between manganese nodules and geoactive fungal species. Our findings provide a reference for future studies in this area and further contribute to an understanding of the applied potential of geoactive fungi in mediating metal and mineral transformations.

## Results

### *Elemental composition of manganese nodules*

The elemental composition of two manganese nodule samples from the same location in the CCZ, Pacific Ocean was determined using X‐ray fluorescence (XRF) spectroscopy (Table 1). It was found that both nodule samples were primarily composed of Mn, with significant amounts of Si, Fe Al, Mg, Na and Ca. These elements made up over 80% of both nodule samples. Further elements that comprised > 1% of both nodule samples were Ni, Cu and K. In COG_MN35, Cl was 1.05% by mass, while in COG_MN12, Cl represented 0.93%. Other elements that were present in both nodules at < 1% included Ti, S, P, Ba, Zn and Co. Elements present at < 0.1% included Mo, Ce and Sr (Table 1).

**Table 1:** Elemental composition of manganese nodule samples.

	COG_MN12	COG_MN35
MnO	43.79	47.25
SiO_2_	19.44	12.05
Fe_2_O_3_	9.42	8.61
Al_2_O_3_	5.82	4.52
MgO	2.99	3.23
Na_2_O	3.12	2.84
CaO	2.67	2.21
NiO	1.44	1.67
CuO	1.51	1.58
K_2_O	1.25	1.11
Cl	0.93	1.05
TiO_2_	0.42	0.46
SO_3_	0.63	0.42
P_2_O_5_	0.44	0.33
BaO	0.80	0.29
ZnO	0.22	0.24
CoO	0.17	0.15
MoO_3_	0.09	0.09
CeO_2_	0.02	0.09
SrO	0.09	0.07
		
Total	95.25	88.24

Manganese nodule composition (% by mass) determined using X‐ray fluorescence (XRF). Samples were compacted under a load of 75 kN for 5 min and 150 kN for 10 min prior to analysis. The results are expressed as oxides.

### *Colonization of manganese nodules by A. niger*

**Figure 1:**
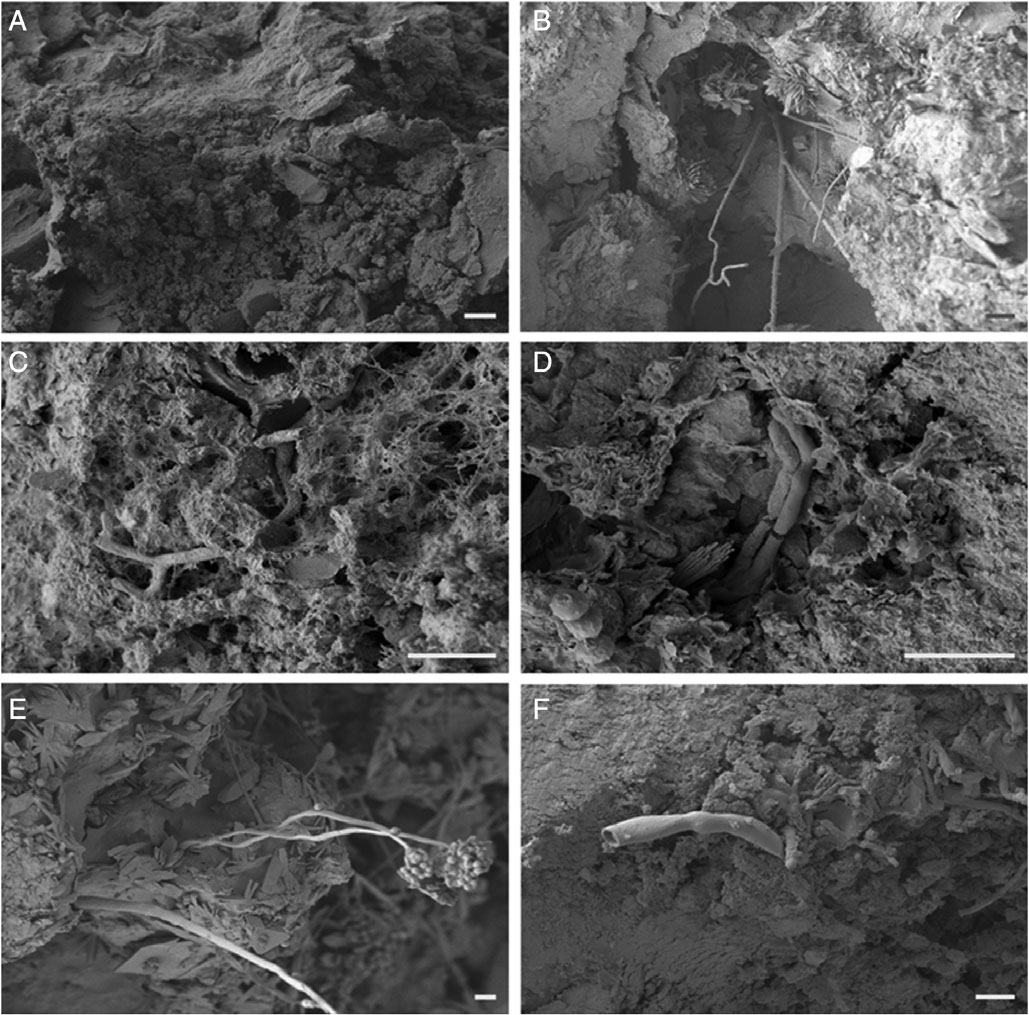
Colonization of manganese nodule fragments by *A. niger*. SEM of colonized COG_MN12 manganese nodule fragments following incubation with either *A. niger* ATCC 1015 (C, E, F) or *A. niger* ΔoafA (B, D). A. Surface of an uninoculated control fragment following 2 weeks incubation in the dark at 25°C, scale bar = 10 μm. B. Branched hyphae emerging from a cavity in the nodule fragment following 1 week of incubation in the dark at 25°C, scale bar = 10 μm. C. Exposed internal surface of a COG_MN12 fragment showing a hypha growing through a fissure following 3 weeks incubation in the dark at 25°C, scale bar = 10 μm. D. Hyphae emerging from and growing into a nodule fragment following 4 weeks incubation in the dark at 25°C, scale bar = 10 μm. E. Conidiophores growing from the surface of a nodule fragment following 2 weeks incubation in the dark at 25°C, scale bar = 10 μm. F. Hypha growing from the surface of a nodule fragment following 1 week of incubation in the dark at 25°C, scale bar = 10 μm. Images shown are representative of several examinations.

**Figure 2:**
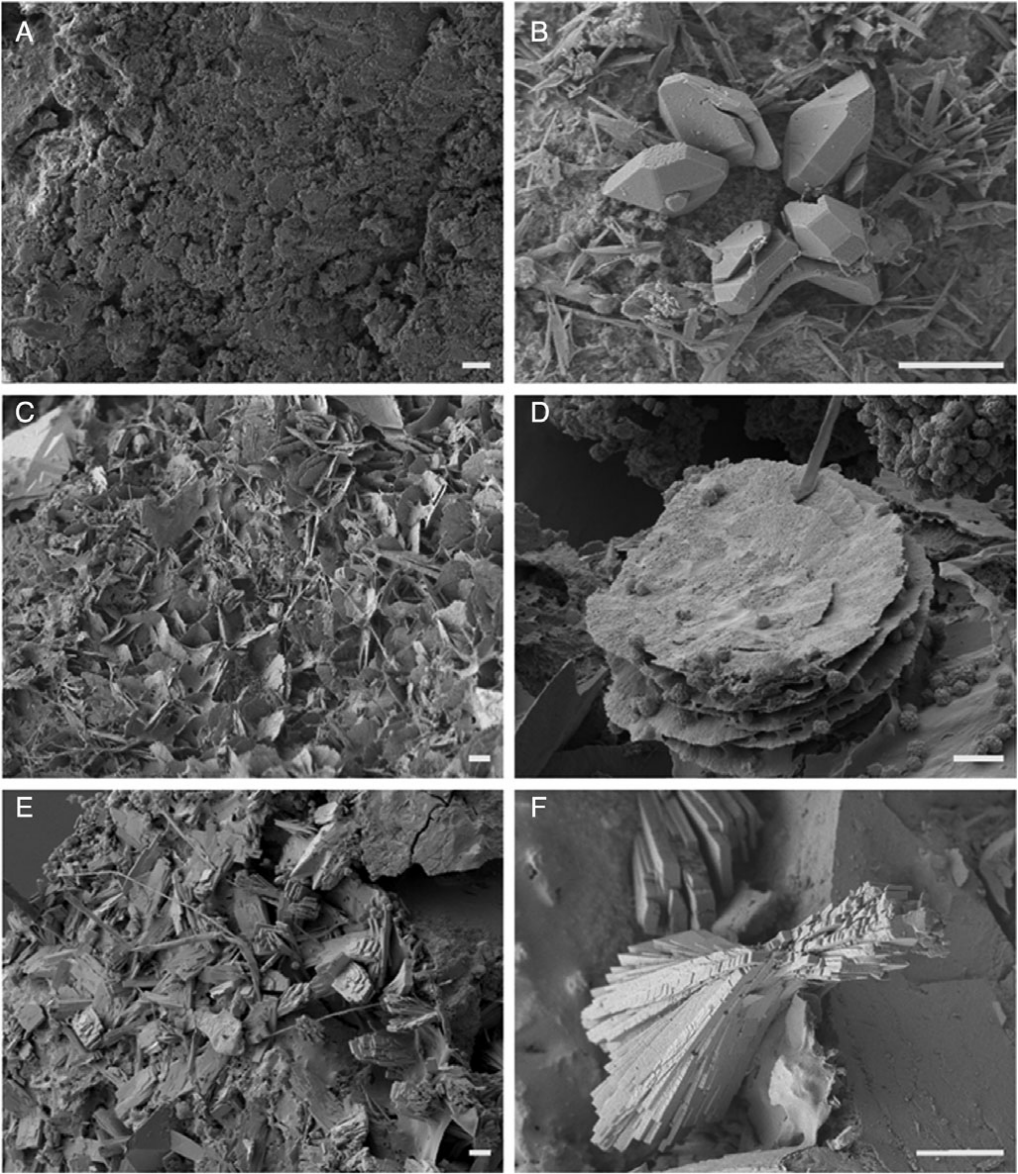
Secondary biominerals formed during manganese nodule fragment colonization by *A. niger*. SEMof biominerals on colonized COG_MN12 manganese nodule fragments following incubation with either *A. niger* ATCC 1015 (B, C, D) or *A. niger* ΔoafA (E, F). A. Surface of uninoculated control fragment following 6 weeks incubation in the dark at 25°C, scale bar = 10 μm. B. Octahedral biominerals observed following 4 weeks incubation in the dark at 25°C, scale bar = 10 μm. C. Crust of shard‐like biominerals observed following 2 weeks incubation in the dark at 25°C, scale bar = 10 μm. D. Shard‐like biominerals in a rosette structure following 2 weeks incubation in the dark at 25°C, scale bar = 10 μm. E. Crust of monoclinic biominerals observed following 1 week of incubation in the dark at 25°C, scale bar = 10 μm. F. Individual monoclinic biomineral observed following 1 week of incubation in the dark at 25°C, scale bar = 10 μm. Incubations were carried out in the dark and images shown are representative of several examinations.

**Figure 3:**
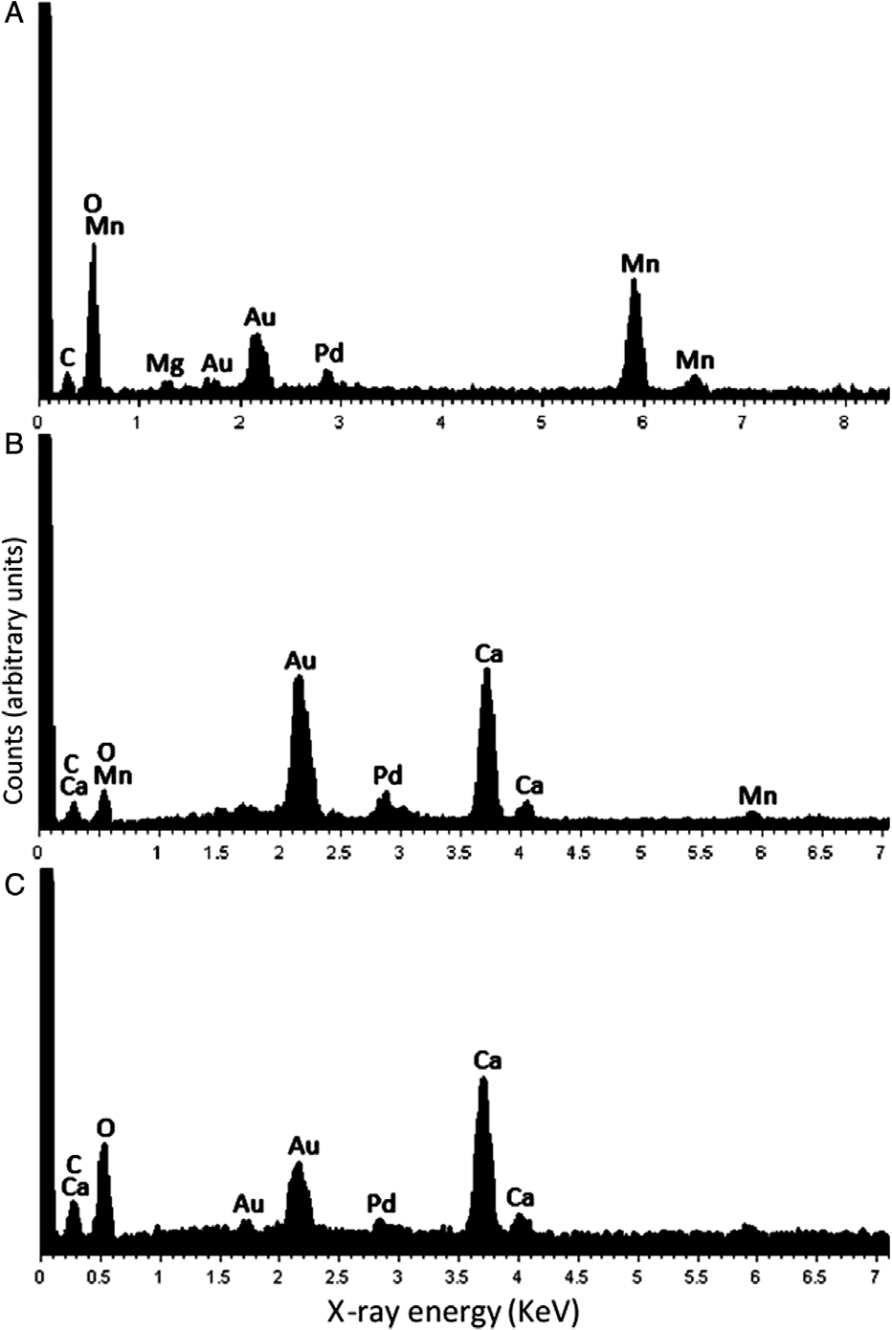
Elemental composition of secondary biominerals occurring on manganese nodules colonized by *A. niger*. EDXA of biominerals observed on COG_MN12 manganese nodule fragments following incubation with either *A. niger* ATCC 1015 or *A. niger* ΔoafA incubated for 2 (A), 1 (B) and 4 (C) weeks at 25°C in the dark. A. Shard‐like biominerals. B. Monoclinic biominerals. C. Octahedral biominerals. Spectra shown are representative of at least three determinations. The gold and palladium peaks are due to the sample coating.

The scanning electron microscopy (SEM) images in Fig. 1 show colonization of COG_MN12 nodule fragments by both *A. niger* ATCC 1015 and *A. niger* ΔoafA. Both strains of *A. niger* were able to successfully colonize the COG_MN12 fragments. Branched hyphae of *A. niger* ΔoafA were observed emerging from cavities in the nodule substrate after 1 week of incubation (Fig. 1B). Hyphae were also observed emerging from cavities and returning to the mineral interior (Fig. 1D). An exposed internal surface is shown in Fig. 1C in which a hypha of *A. niger* ATCC 1015 can be seen to have grown through a fissure following 2 weeks of incubation. Additionally, hyphae (Fig. 1F) and conidiophores (Fig. 1E) of *A. niger* ATCC 1015 grew from the nodule surface after 1 and 2 weeks of incubation respectively. Secondary mineral formation was frequently observed surrounding the sites of fungal colonization (Fig. 1B, D, and E). Fig. 2 shows examples of secondary biomineral formation observed through SEM examination of COG_MN12 fragments colonized by either *A. niger* ATCC 1015 or *A. niger* ΔoafA. Octahedral biominerals were frequently observed (Fig. 2B) while shard‐like secondary mineral formations were observed as either crusts (Fig. 2C) or individual rosettes (Fig. 2D). Monoclinic formations were observed that were present as both crusts (Fig. 2E) and individual structures (Fig. 2F). Energy dispersive X‐ray analysis (EDXA) revealed that the biominerals observed were primarily composed of either manganese or calcium, with carbon and oxygen. Figure 3A shows a typical EDXA spectrum obtained from the shard‐like formations showing strong peaks corresponding to manganese. Strong peaks for calcium, carbon and oxygen were found in the EDXA spectra for the octahedral and monoclinic biomineral morphologies (Fig. 3B and C).

### *Transformation of manganese nodules by A. niger ATCC 1015*

**Figure 4:**
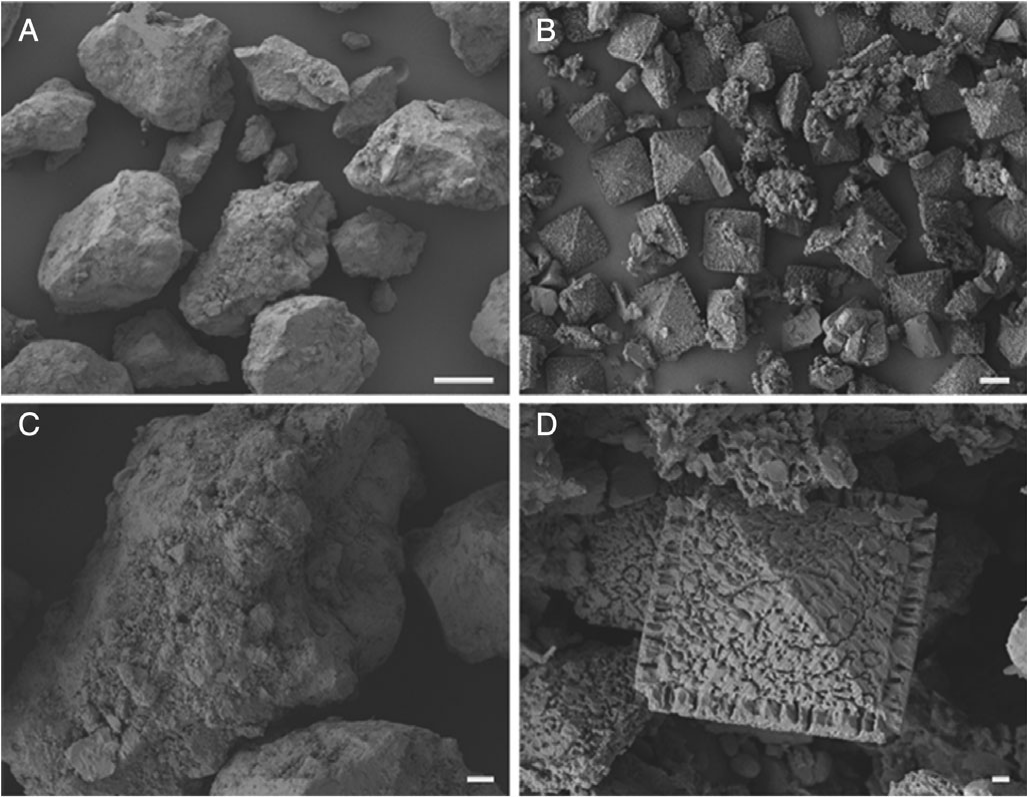
Transformation of manganese nodule powder by *A. niger*. SEM of transformed manganese nodule powder following 2 weeks incubation in the dark at 25°C with *A. niger* ATCC 1015. A. Uninoculated control powder recovered from MEA following 2 weeks incubation in the dark at 25°C, scale bar = 100 μm. B. Biomineral debris recovered from MEA plates containing manganese nodule powder at 0.2% (w/v) following 2 weeks incubation in the dark at 25°C with *A. niger* ATCC 1015, scale bar = 10 μm. C. Uninoculated control powder recovered from MEA following 2 weeks incubation in the dark at 25°C, scale bar = 10 μm. D. Octahedral biomineral recovered from MEA plates containing manganese nodule powder at 0.2% (w/v) following 2 weeks incubation with *A. niger* ATCC 1015 in the dark at 25°C, scale bar = 1 μm. Typical images from several examinations are shown.

**Figure 5:**
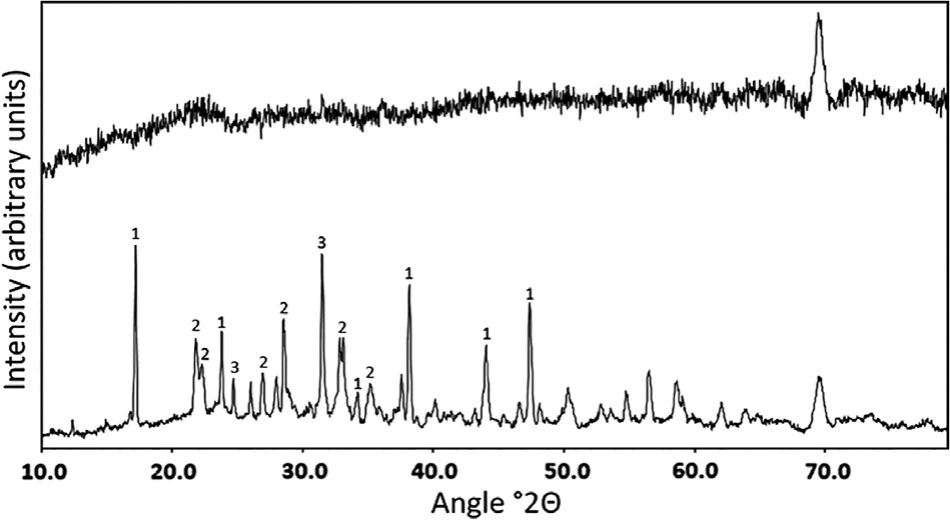
Mineralogical analysis of manganese nodule powder transformed by *A. niger*. X‐ray diffraction patterns of mineral debris recovered from MEA plates amended with manganese nodule powder at 0.2% (w/v). Diffraction patterns were obtained from (A) uninoculated control plates and (B) plates inoculated with *A. niger* ATCC 1015 following 2 weeks incubation at 25°C in the dark. (i) Pattern of calcium oxalate dihydrate, weddellite. (ii) Pattern associated with manganese oxalate dihydrate, lindbergite. (iii) Pattern associated with silicon oxide, quartz. Typical patterns are shown from one of at least three determinations.

As the nodule fragment colonization studies had shown no observable difference between the *A. niger* strains only the wild‐type, *A. niger* ATCC 1015, was used further. Almost complete transformation of manganese nodule powder incorporated into MEA occurred following 2 weeks incubation with *A. niger* ATCC 1015 (Fig. 4). The bulk of the resulting mineral debris was composed of octahedral biomineral structures ranging from approximately 10 to 30 μm in diameter, with smaller amorphous debris (Fig. 4B and D). X‐ray diffraction (XRD) analyses of the biomineral debris recovered from MEA plates containing 0.2% (w/v) COG_MN35 powder, following 2 weeks incubation with *A. niger* ATCC 1015, confirmed the presence of calcium oxalate dihydrate (weddellite) and manganese oxalate dihydrate (lindbergite) (Fig. 5B). The pattern produced by the transformed mineral was in clear contrast to that obtained from the poorly crystalline control phase that was not exposed to fungal interactions (Fig. 5A).

### *Manganese nodule interactions with A. niger ATCC 1015 in liquid media*

**Figure 6:**
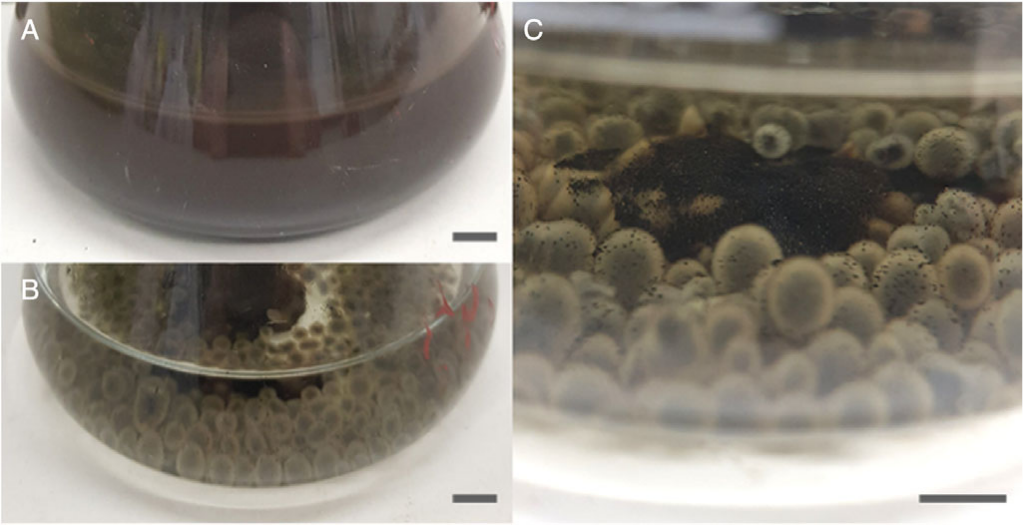
Transformation of manganese nodule powder by *A. niger* in liquid medium. Images show comparison of (A) an uninoculated control flask containing AP1 medium amended with 2% (w/v) COG_MN35 powder and (B) a flask inoculated with 10^6^ spores ml^−1^ of *A. niger* ATCC 1015 and incubated for 6 days at 25°C with shaking at 125 rpm. (C) COG_MN35 powder within the pellets. Scale bars = 1 cm.

**Figure 7:**
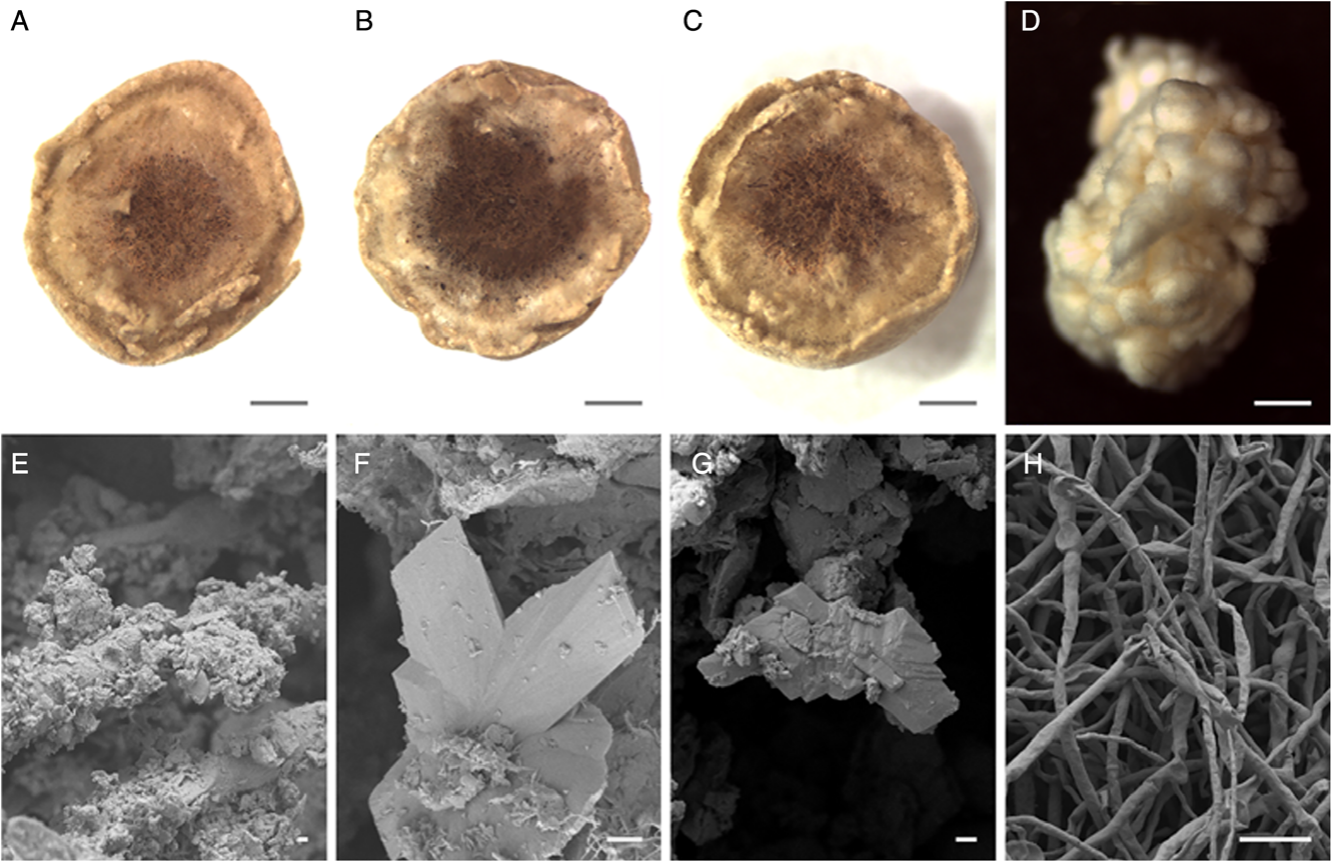
Accumulation and transformation of manganese nodule powder within *A. niger* pellets. Light microscopy and SEM images of *A. niger* ATCC 1015 pellets grown in liquid AP1 medium amended with 2%(w/v) COG_MN35 powder and recovered after 14 days incubation at 25°C with shaking at 125 rpm. A, B, C. Cross sections of *A. niger* pellets obtained by light microscopy following 2 weeks incubation at 25°C with shaking at 125 rpm. D. *A. niger* pellet recovered from control medium following 14 days incubation in AP1 medium without manganese nodule powder. E. SEM image of *A. niger* hyphae with a crust of mineral debris obtained from the central region, scale bar = 1 μm. F, G. SEM images of biomineral components of the mineral crust surrounding *A. niger* hyphae, scale bar = 1 μm. H. SEM image of *A. niger* hyphae obtained from the control biomass, scale bar = 10 μm. Typical images are shown from one of several examinations.

**Figure 8:**
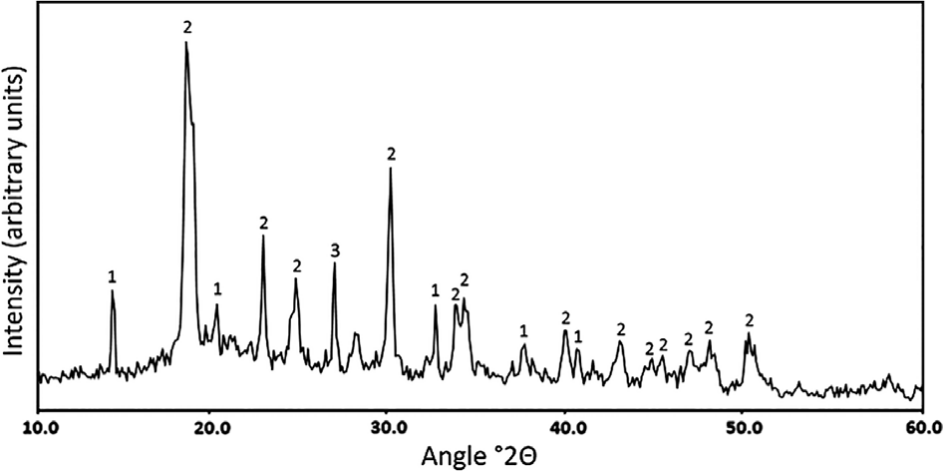
Mineralogical analysis of manganese nodule debris within *A. niger* pellets. X‐ray diffraction patterns of powdered *A. niger* ATCC 1015 pellets grown in AP1 medium amended with 2% (w/v) COG_MN35 powder and incubated for 2 weeks at 25°C with shaking at 125 rpm. (i) Patterns associated with calcium oxalate monohydrate, whewellite. (ii) Patterns associated with manganese oxalate dehydrate, lindbergite. (iii) Patterns associated with quartz. Typical patterns are shown from one of several examinations.

In liquid media, the addition of COG_MN35 manganese nodule powder resulted in a black turbid suspension. However, media clarity improved markedly over the course of the incubation, and incorporation of the nodule powder into the fungal pellets was observed (Fig. 6). The nodule powder accumulated within the central region of the mycelial pellet closely associating with the hyphae (Fig. 7A–C). SEM examination of the pellet central regions revealed crusts of densely organized amorphous mineral debris forming sheaths around the hyphae (Fig. 6E). Small, angular secondary biomineral structures up to ~10 μm in diameter were also frequently observed as a component of these mineral sheaths (Fig. 7F and G). Control *A. niger* biomass displayed loosely organized growth and did not form robust spherical pellets of the kind observed in the presence of manganese nodule powder (Fig. 7D and H). X‐ray diffraction analysis of dried and powdered *A. niger* ATCC 1015 pellets, following 2 weeks incubation, revealed patterns with a match to reference patterns corresponding to calcium oxalate dihydrate and manganese oxalate dihydrate (Fig. 8).

## Discussion

Recent years have seen unprecedented increase in demand for raw materials to meet the growing need for infrastructure and manufacturing. This is further compounded by the diverse range of elements needed for the manufacture of current technology, which makes use of a number of metals that have no or few direct primary sources. Such metals are often produced as by‐products in the production of primary metals, such as cobalt recovery from copper mining, resulting in supply chains that are therefore vulnerable to factors that influence primary metal production. Due to their economic importance, vulnerable supply chains and the lack of viable substitutions for their application these metals are often termed ‘critical’ (Graedel *et al.,* 2015; Wall *et al.,* 2017). As a consequence, much attention has been focused on improving the supply chain for critical metals through the identification of new mineral resources and the development of novel processing strategies, with a particular emphasis on low‐carbon processing due to the role of many critical metals in green energy technologies. This research is carried out as part of the CoG^3^ consortium, a Natural Environment Research Council (NERC, UK) initiative dedicated to improving understanding of the geology, geometallurgy and geomicrobiology of cobalt, in a drive to improve the security of supply of this element. Manganese nodule resources represent a wealth of metals with wide ranging uses as both raw materials for infrastructure, such as Cu, Ni and Mn, and critical metals with application in advanced electronic and energy technologies, including Co, Nb and several REE (Hein *et al.,* 2013; Vidal *et al.,* 2017). Bioprocessing strategies using bacteria and fungi have received renewed attention in this context as efficient low‐cost and low‐carbon alternatives to traditional metal processing approaches (Strasser *et al*., 1994; Mulligan and Galvez‐Cloutier, 2000; Mulligan *et al.,* 2004; Biswas *et al.,* 2013; Zeng *et al.,* 2015; Jadhav *et al.,* 2016; Liang and Gadd, 2017). In the light of these trends, we have investigated some fundamental interactions between manganese nodules and *Aspergillus niger*, a geoactive species considered to be particularly well suited to such applications.

In this research, the ability of *A. niger* to colonize and transform manganese nodules from the Clarion‐Clipperton Zone (CCZ) was clearly demonstrated in both solid and liquid media. Both strains of *A. niger* (ATCC 1015 and ΔoafA) were able to colonize manganese nodule fragments and fungal hyphae were observed emerging from cavities in the nodule fragments at locations distant from the sites of primary colonization. When considering additional observations of hyphae growing through and across fissures of exposed internal faces, it can be concluded that the fungus was able to access the interior of the nodule fragments. This is in line with current understanding of the growth strategies of many fungal species, which can employ explorative growth in rock and mineral substrates. Such a strategy is well suited to porous substrates with high metal concentrations in immobile forms, such as manganese nodules. Furthermore, mycelial fungi are known to be able translocate nutrients between regions in heterogeneous environments, which enables them to colonize otherwise inhospitable substrates (Olsson, 1999; Fomina *et al.,* 2003; Boswell *et al.,* 2007). Biomineral formation was frequently observed close to the sites of fungal colonization suggesting that metabolite secretion, especially organic acids, has played an important role in such a biotransformation. *Aspergillus niger* has a well‐established capacity for organic acid production, and mycogenic organic acid metabolites are a major contributor to bioweathering in free‐living and symbiotic fungi. Fungal hyphae are known to penetrate into weatherable mineral phases and organic acids may facilitate such activity (Gadd, 2014; 2007; 2017a,b). In this study, the exact contribution of organic acids in penetration of the manganese nodule substrate is unclear, however, in relation to the exploitation of pre‐existing cracks and fissures, it is likely that both biochemical and biophysical mechanisms played a role in colonization and transformation.

The biominerals occurring on the surface of colonized manganese nodule fragments were of three distinct morphologies: shard‐like, monoclinic sheets and octahedral (eight‐faced bi‐pyramidal). Shard‐like and monoclinic morphologies formed large biomineral crusts, and shard‐like biominerals were also observed forming rosette‐shaped structures. Apart from carbon and oxygen, EDXA revealed that the metal component was calcium in the monoclinic and octahedral biominerals, while the shard‐like morphologies contained manganese. Based on the presence of calcium in the octahedral biominerals and their distinct morphology, these were assumed to be weddellite, a well characterized calcium oxalate dihydrate mineral that is regularly observed in association with many fungi (Heijnen and Vanduijneveldt, 1984; Gadd, 1999; Jarosz‐Wilkolazka and Gadd, 2003; Burford *et al.,* 2006; Gadd *et al.,* 2014). It can be hypothesized that the monoclinic biominerals observed were whewellite, the monohydrated form of calcium oxalate (Gadd, 1999; Gadd *et al.,* 2014). The manganese nodules contain significant concentrations of calcium, and calcium oxalate is frequently associated with calcium‐bearing minerals following interaction with fungi (Burford *et al.,* 2006). The manganese‐containing biominerals were likewise presumed to be manganese oxalates. This matches previous studies which found that *A. niger* was able to transform a range of manganese oxides to manganese oxalates, including birnessite (Wei *et al*., 2012), an ordered phyllomanganate found in many manganese nodules (Hein *et al.,* 2013; Kuhn *et al.,* 2017).

To confirm the mineralogy of the biominerals formed, manganese nodule transformations were also carried out in solid and liquid media. Extensive transformation of the manganese nodule powder was observed in solid media and SEM revealed that the mineral debris consisted primarily of octahedral biominerals, with only small amounts of amorphous material. This provided strong supporting evidence that organic acids generated by *A. niger* were able to transform the manganese nodule powder. This was confirmed by XRD analysis that confirmed the presence of calcium oxalate dihydrate (weddellite, CaC_2_O_4_∙2H_2_O) and manganese oxalate dihydrate (lindbergite, MnC_2_O_4_∙2H_2_O). There was a marked contrast in XRD patterns between control and inoculated samples, with clear diffraction patterns only observed for the latter. It is known that the fine grain size of manganese nodules can cause difficulties in achieving clear XRD patterns, which further emphasizes the extent of fungal transformation achieved (Johnson and Glasby, 1969; Post, 1999). XRD analysis only showed the presence of weddellite in the transformed nodule powder, and monoclinic calcium oxalate biominerals characteristics of whewellite were not observed.

Following incubation in liquid media, significant accumulation of mineral powder occurred within the fungal pellets resulting in almost complete clarification of the original turbid black suspension. Light microscopy revealed dense aggregates of mineral debris and hyphae forming distinct dark‐coloured regions at the centre of the pellets. SEM examination of pellet central regions confirmed that this was composed of mineral crusts around the hyphae composed primarily of small amorphous particles, with angular mineral structures also being observed. When considering the obvious difference in media clarity between uninoculated control flasks and those containing mycelial pellets, it can be concluded that during the initial growth period, fine particulates in suspension were bound to and trapped by the growing hyphae. The mycelium would then continue to grow around the mineral‐bearing region. XRD analysis of dried and powdered pellets again showed the presence of manganese and calcium oxalates, confirming that *A. niger* was able to transform the manganese nodule substrate in liquid media as a result of oxalic acid excretion.

The capacity of fungal biomass in sorbing a variety of substances is well established (Gadd, 2009). For example, biomass of *Aspergillus* spp. has been demonstrated to bind several metals including Mn, Co, Ni, Zn and Cu (Akthar *et al.,* 1996; Baik *et al.,* 2002; Parvathi *et al.,* 2007; Mukhopadhyay *et al.,* 2008). Such binding of metal ions is largely a function of the diverse range of reactive functional groups on fungal cell walls, including carboxyl (—COOH), thiol (—SH), hydroxyl (—OH), carbonyl (—C=O) and methoxyl (—O—CH_3_) groups. Furthermore, the amine functional groups in chitin (R_2_—NH) contribute to metal binding, as well as functional groups in other cell wall polymers such as melanin (Gadd, 2009; Fomina and Gadd, 2014). Metal sorption on fungal hyphae can also act as nucleation sites for extracellular metal precipitation, enhancing metal removal (Burford *et al.,* 2006; Gadd, 2007; 2009; 2010). Importantly, and particularly relevant to this study, fungi have also received attention for the separation of particulate matter from solution, such as the removal of activated charcoal (Wainwright, 1999). Fomina and Gadd (2002) were able to take advantage of the particulate binding properties of fungal biomass for the production of composite mycelial pellets containing clay minerals as efficient toxic metal sorbents. In conclusion, our work represents the first examples of manganese nodule transformations by fungi. The results extend the understanding of the possible interactions between fungi and manganese‐containing mineral substrates that are widespread in the biosphere, although the significance of such transformations in the natural manganese nodule deep sea environment is a matter for conjecture. Nevertheless, future work on the deep sea microbial consortia colonizing such substrates may reveal hitherto undiscovered geomicrobial processes as has been the case for other deep subsurface locations (Ivarsson *et al*., 2015, 2016a,b). Our findings may also have practical significance for future mineral bioprocessing applications using fungi, with additional relevance to environmental processes involving chemoorganotrophic microorganisms and manganese minerals.

## Experimental procedures

### *Organisms, growth conditions and media*

The organisms used were *A. niger* (ATCC1015) and *A. niger* ΔoafA (kindly provided by Dr J. Thykær, Technical University of Denmark, Lyngby, Denmark) and were maintained on malt extract agar (LabM, Bury) at 25°C in the dark. Malt extract agar (MEA) (Sigma‐Aldrich, St. Louis, MO) was used for solid media experiments. AP1 medium was used for liquid media experiments and contained the following (mmol l^−1^ Milli‐Q water): 111.01 D‐glucose, 37.86 (NH_4_)_2_SO_4_, 36.74 KH_2_PO_4_, 0.81 MgSO_4_·7H_2_O, 0.23 CaCl_2_·6H_2_O, 1.71 NaCl, 9.25 × 10^−3^ FeCl_3_·6H_2_O. The following trace metals were also included: 1.39 × 10^−8^ ZnSO_4_·7H_2_O, 1.79 × 10^−8^ MnSO_4_·4H_2_O and 1.6 × 10^−9^ CuSO_4_·5H_2_O. All chemicals were obtained from Sigma‐Aldrich unless stated otherwise.

### *Manganese nodule transformations in solid and liquid media*

Manganese nodules (kindly provided by Professor S. Roberts, National Oceanography Centre, University of Southampton, Southampton, SO14 3ZH, UK) were ground into fragments and powder using a pestle and mortar. Powdered samples were sieved to a particle diameter of < 210 μm or < 90 μm using the appropriate soil sieves (Endecotts, London, SW19 3TZ, UK). Samples were sterilized in an oven at 105°C for at least 48 h prior to experimentation. Manganese nodule amended solid media was prepared by incorporating sterile COG_MN35 powder of particle diameter < 210 μm into molten MEA at ~60°C to a final concentration of 0.2% (w/v) and allowing to set at room temperature. Fragment colonization experiments were set up by melting specific locations in MEA plates with stainless steel forceps heated to ~90°C using a Bunsen burner and inserting 6× COG_MN12 fragments approximately 125 mm^3^ into the resulting wells. Six fragments were used per Petri dish, places equidistantly apart and 3 cm from the plate centre. Solid media was centrally inoculated using a 5 mm diameter disc of *A. niger* ATCC 1015 mycelium taken from the edge of an actively‐growing colony on MEA using a sterile cork borer. Manganese nodule amended liquid media was prepared by adding sterile COG_MN35 powder of particle diameter < 90 μm to AP1 medium to a final concentration of 2% (w/v). The finer particle size was chosen in order to maintain the mineral powder in suspension and minimize the sedimentation. Liquid media was inoculated using 1 ml of an *A. niger* spore suspension containing 10^6^ spores, giving a final concentration of 10^4^ spores ml^−1^. Spore suspensions were prepared by adding approximately 20 ml 0.2% TWEEN 80 to an *A. niger* slant grown on MEA for approximately 1 h at room temperature. The slant was shaken vigorously to suspend spores, before filtering the suspension through sterile miracloth (pore size 22–25 μm) to remove mycelium (MilliporeSigma, Burlington, MA). The suspension was washed three times with sterile Milli‐Q water by centrifuging at 1000 rpm (101 g) for 15 min before resuspending the spores in 20 ml Milli‐Q water. Spore concentrations were determined using a Neubauer chamber (Weber Scientific, Hamilton, NJ) and the suspension diluted to a final concentration of 10^6^ spores ml^−1^ using sterile Milli‐Q water.

### *Recovery of minerals from manganese nodule amended solid media*

Following 14 days incubation at 25°C with shaking at 125 rpm, the biomass was carefully removed using a scalpel and the minerals and debris extracted by gently homogenizing the agar in Milli‐Q water heated to approximately 70°C. Mineral debris were then washed twice with Milli‐Q water heated to approximately 70°C and dried in a desiccator for 1 week prior to analyses.

### *Analysis of mineral powder, fragments and mycelial pellets*

#### *Scanning electron microscopy and energy dispersive X‐ray analysis*

Colonized fragments from solid media and *A. niger* pellets from liquid media were fixed for ~24 h in 2.5% (v/v_aq_) glutaraldehyde solution in 5 mM piperzine‐N,N′‐bis (2‐ethanesulfonic acid) (PIPES) buffer adjusted to pH 6.5 with 1 M NaOH. Samples were washed twice with 5 mM PIPES buffer, pH 6.5, before dehydration using an ascending ethanol series [30%–100% (v/v_aq_)], with the samples being left for 10 min at each step. Samples were then dried using a CO_2−_ critical point drier (BAL‐TEC CPD 030; Bal‐Tec company, Los Angeles, CA). Dried samples were mounted on double‐sided adhesive carbon tape on 25 mm × 5 mm aluminium electron microscopy stubs and coated with 15 nm gold and palladium using a Cressington 208HR sputter coater (Cressington Scientific Instruments, Watford, UK). Samples were examined using a field emission scanning electron microscope (JEOL JSM‐7400F) operating at an accelerating voltage of 5 kV for imaging and 20 kV for EDXA.

#### *XRF spectroscopy*

Partial elemental composition was determined for the manganese nodule samples by XRF spectroscopy using a Philips ‘Zetium’ PW5400 sequential X‐ray fluorescence spectrometer with a RhKα source (Malvern Panalytical, Malvern, UK). Samples were compacted under a load of 75 kN for 5 min then 150 kN for 10 min before analysis. The results are expressed as oxides.

#### *X‐ray diffraction*

Mineralogical analysis was carried out using X‐ray powder diffraction (XRPD) on an Enraf‐Nonius FR590 powder diffraction system (Enraf‐Nonius, Rotterdam, the Netherlands). Samples were prepared by grinding using a mortar and pestle before applying to a quartz substrate as an acetone smear. Diffraction patterns were recorded from 0 to 80° 2‐theta using an INEL 120° curved position sensitive detector, with Co K‐alpha radiation. The samples were continuously spun in the plane of the sample surface for a 2 h data collection period. Phases were identified with reference to patterns from the International Centre for Diffraction Data (ICDD) Powder Diffraction File (PDF) database.

#### *Light microscopy*

Light microscopy was conducted using a Leica EZ4 HD stereo microscope with LED illumination, a parfocal optical system and 60° viewing angle (Meyer Instruments Houston, TX). Images were obtained using the integrated 3.0 Mega‐Pixel CMOS camera and processed using Leica LAS EZ software (Meyer Instruments).
